# A Numerical Model to Predict the Relaxation Phenomena in Thermoset Polymers and Their Effects on Residual Stress during Curing, Part II: Numerical Evaluation of Residual Stress

**DOI:** 10.3390/polym16111541

**Published:** 2024-05-30

**Authors:** Raffaele Verde, Alberto D’Amore, Luigi Grassia

**Affiliations:** Department of Engineering, University of Campania “Luigi Vanvitelli”, via Roma, 29, 81031 Aversa, Italy; raffaele.verde1@unicampania.it (R.V.); alberto.damore@unicampania.it (A.D.)

**Keywords:** epoxy, viscoelasticity, cure, structural relaxation, residual stresses, numerical simulation

## Abstract

This article proposes a numerical routine to predict the residual stresses developing in an epoxy component during its curing. The scaling of viscoelastic properties with the temperature and the degree of conversion is modeled, adopting a mathematical formulation that considers the concurrent effects of curing and structural relaxation on the epoxy’s viscoelastic relaxation time. The procedure comprises two moduli: at first, the thermal–kinetical problem is solved using the thermal module of Ansys and a homemade routine written in APDL, then the results in terms of temperature and the degree of conversion profiles are used to evaluate the viscoelastic functions, and the structural problem is solved in the mechanical module of Ansys, allowing the residual stresses calculation. The results show that the residual stresses mainly arise during cooling and scale with the logarithm of the Biot number.

## 1. Introduction

Since epoxy has unique properties in adhesion, strength, toughness, and processability, it is used in many different sectors nowadays. Epoxy’s components are utilized in electric and electronic applications as packaging for electronic devices [1,2], adhesives [3,4], coating [5], and so on [6,7]. In addition, epoxy resins are the most used matrices for carbon and glass fiber composites, employed mainly in aerospace [8,9].

From the processing point of view, thermosets are generally made of an initial liquid mixture of polyfunctional organic molecules that gradually react to form a three-dimensional crosslinked molecular network. There are many kinds of epoxy resins, the types and the number of constituents and curing agents are chosen to obtain different thermal and mechanical properties for their particular application [6,10]. In addition, the process parameters strongly affect the final thermosetting system behavior in terms of mechanical properties and thermal stability; generally, a high curing temperature provides a higher final glass transition temperature, greater tensile strength, and more significant heat and chemical resistance but lower flexibility and lower impact resistance [6].

During the curing, the resin’s crosslinking reaction is induced by applying external stimuli (generally a change in temperature or UV radiation), allowing the gradual transformation of epoxy from a liquid to an almost solid-like material. During the process, many concurrent phenomena act and influence each other. The most important ones are the thermal expansion/contraction due to positive/negative temperature variation, the chemical reaction of curing, the viscoelasticity, and the structural relaxation. These physical phenomena are all correlated, and the mathematical modeling of their interconnection was performed in various studies [11,12,13,14,15].

Generally, at the end of the curing, polymers, and polymeric composite components show part warpage and distortions [16] originating from residual stresses arising during manufacturing [1,17,18]. These issues often reduce the theoretical mechanical properties and necessitate further re-manufacturing, which can be expensive in terms of both costs and time. In some instances, components show local fractures at the end of the manufacturing, even before any structural loads are applied. In other cases, component failures may occur under structural load significantly lower than the component’s nominal strength, due to a high residual tensional state.

Quantifying the residual stresses and understanding their origin is essential to improving the quality and mechanical properties of polymeric and polymer-based composite parts and optimizing process parameters.

The cause of residual stress in polymers is the development of non-uniform free strains. The free strains are due to non-mechanical loads acting on the system. They are generally produced by thermal variation and phase changes, namely the structure’s shrinkage during crosslinking or crystallization in semicrystalline polymers [19].

In an isotropic and homogeneous body, the development of free strains does not affect the stress in the absence of external constraints. Residual stresses arise in anisotropic free-standing materials due to the material’s direction-dependent properties, namely the coefficient of thermal expansion and the elastic moduli. In that case, the mismatch of free strains could generate significant residual stresses. However, any constrained body can develop residual stresses when subjected to thermal loads.

Residual stresses in polymers and polymer-based composites can be measured experimentally rather than numerically. Experimental techniques allowing residual stress estimation can be divided into two categories: directly measuring the amount of stress during the manufacturing and measuring the stress after the process (interferometry [20], Raman spectroscopy [21], electrical conductance [22]). Direct residual stress detection implies the application of sensors (strain gauges or optic fibers) that can monitor in-situ the evolution of stressors during the manufacturing process [23,24]; they have the limits that the sensors are temperature sensitive and not able to resist high processing temperatures and sometimes sensor debonding occurs (especially at a low degree of conversion).

The post-processing residual stress evaluation can be distinguished between non-destructive and destructive techniques [25]. These techniques have limitations and can be used only in particular cases: interferometry can only measure superficial stress, while Raman spectrometry needs a transparent material. Hole drilling is a destructive test that, upon removing a portion of the material, measures the relieved strains with rosette strain gauges [20,26,27].

All of the experimental techniques have limitations; they can be used only in particular situations, and in many cases, their application is prohibitive in terms of costs and time [25]. To overcome all the difficulties due to experimental techniques, using a numerical model that virtually simulates the manufacturing process could be beneficial to understanding the magnitude of residual stresses. It is crucial to accurately model a material’s mechanical and physical behaviors to achieve plausible results. Since the crosslinking reaction causes a change in the molecular structure, the first step involves modeling how the degree of conversion affects the mechanical properties. The curing process, in fact, increases the glass transition temperature and the viscoelastic relaxation time. Various models have been developed to understand how the curing process influences the mechanical behavior of epoxy. In many simplified models [28,29,30,31,32], elastic behaviors were simulated and an empirical dependence of the elastic modulus on the temperature and the degree of conversion is assumed. One of the first models addressing this topic was proposed by Bogetti et al. [32]: they hypnotized that elastic behaviors and the epoxy modulus is a function of the degree of curing. This formulation was subsequently improved by also incorporating the dependence on temperature as reported in the CHILE (Cure Hardening Instantaneous Linear Elastic) model [29]. These models have the advantage of simplicity in modeling and FE implementation, but they cannot consider the effect of stress relaxation due to the viscoelastic nature of polymers at elevated temperatures, and in many cases overestimate the stress levels.

Kim and White proposed a viscoelastic model [33] that can be considered a “gold standard” and was often adopted as a benchmark to evaluate simplified models [7]. They conducted experiments by curing epoxy coupons until reaching different conversion degrees. Then, the shear relaxation master curves were experimentally evaluated at different degrees of curing using DMA equipment, and an expression for the time–temperature degree of conversion shift factor was proposed.

Based on these experimental data, in part I [34] of this article, the authors have proposed a mathematical formulation for the shift factor that considers the concurrent effect of curing and structural relaxation on the epoxy’s viscoelastic times for the first time. This expression was then implemented in finite elements software to simulate the polymer’s relaxation phenomena in terms of stress relaxation, creep, and fictive temperature relaxation at different temperatures and degrees of conversion.

Many researchers have studied the problem of residual stresses due to the curing process. In particular, the authors [35,36] have previously investigated the distortions and residual stresses in an amorphous polymer during its cooling from a liquid to a glassy state. They analyzed two geometries (an axial symmetrical cylinder and a cantilever beam) subjected to non-uniform thermal history, and in both cases, the residual stresses are relevant. It was assured that the volume change due to structural relaxation plays a crucial role in developing stress.

In another work [37], they investigated the residual stresses in a UV-cured thermoset composite used for dental restoration. In that case, the origins of stress are due to the free strain produced by the thermal change, the crosslinking reaction, and the mechanical constraints between the restoration and the tooth. Different geometries were analyzed; in some cases, the residual stress level can exceed the material’s nominal strength. The dental composite was damaged at its interface as soon as it was made, reducing its durability.

Generally, these stress entities are more consistent in a two-phase component than in epoxy only components due to the thermal and mechanical properties mismatching between the two phases [17,28,38,39]. In addition, in a polymer-based composite laminate, the orthotropy of ply causes an in-thickness mismatch of mechanical and thermal properties, such as angle-ply laminate, that produces distortions and residual stresses in the composite part.

In this article, we apply the theoretical model introduced in part I of this work [34] to numerically estimate the internal stresses that develop within an epoxy cylinder when subjected to a generic thermal history. The aim is to evaluate the internal stresses without using experimental techniques and understand the factors influencing them to manage them during manufacturing better, thereby enhancing product quality. Compared to other models in the literature, the proposed formulation considers the relaxation phenomena due to the viscoelastic nature of polymers and the concurrent effect of curing and structural relaxation on epoxy viscoelastic times.

## 2. Materials and Methods

### 2.1. Mathematical Formulation

The mathematical formulation to model the curing process was proposed in part I of this work [34], summarized here.

The main parameter that identifies the state of the curing process is the degree of conversion, defined as:

(1) $$\alpha =\frac{H(t)}{H_{r}}$$

where H(t) is the internal heat produced at time t by the curing reaction, and H_r_ is the total heat produced at the end of the reaction.

The rate of conversion is a function of temperature and time. In the framework of this modeling approach, the kinetics model proposed by Bogetti et al. [40] is used:

(2) $$\frac{d\alpha }{dt}=(k_{1}+k_{2}\;\alpha )\left(0.47-\alpha \right)\;\;\;\;\;\;\;\;\;for\;(\alpha \leq \;0.3)$$

(3) $$\frac{d\alpha }{dt}=k_{3}\left(1-\alpha \right)\;\;\;\;\;\;\;\;for\;\left(\alpha \geq \;0.3\right)\;\;\;\;\;\;\;\;\;\;\;\;\;\;\;\;\;\;\;\;\;\;$$

k_1_, k_2_ and k_3_ follow the Arrhenius law:

(4) $$k_{i}=A_{i}\;Exp\left(-\frac{{∆E}_{i}}{RT}\right)\;\;\;\;\;i=1,2,3\;\;\;\;\;\;$$

where A_i_ (i = 1, 2, 3) are material coefficients, T is the absolute temperature, R is the gas universal constant and Δ*E*_i_ are the activation energies.

The curing is an exothermic process, and the internal heat Q is assumed to be proportional to the rate of conversion:

(5) $$Q=\rho \;H_{r}\frac{\partial \alpha }{\partial t}\;\;\;\;\;\;\;$$

where ρ is the material density, and *H_r_* is the heat generated per unit mass. Thermal–kinetic parameters are reported in Table 1.

The reaction rate is a temperature-activated phenomenon, thus influenced by the exothermic process. Consequently, the thermal profiles and the cure kinetics are solved coupled.

The constitutive equation for linear viscoelasticity is adopted in this case, and the stress–strain formula can be expressed as:

(6) $$\sigma_{ij}=\int_{0}^{\xi }G(\alpha ,\xi -\xi^{\prime })\frac{de_{ij}}{d\xi^{\prime }}\;\;{d\xi }^{\prime }+\delta_{ij}\int_{0}^{\xi }\frac{1}{3}\;K(\alpha ,\xi -\xi^{\prime })\frac{d(\varepsilon -\varepsilon_{free})}{d\xi^{\prime }}\;\;{d\xi }^{\prime }$$

where σ_ij_ and ε_ij_ = e_ij_ + (1/3) ε are the components of the stress and strain tensors, respectively. δ_ij_ is the Kronecker delta.

*ε_free_* is the free strain due to the non-mechanical loads applied to the system. In the case under study, it is the sum of thermal and chemical strains. The chemical strain considers the contraction due to crosslinking network formation.

G and K are the shear and the bulk relaxation modulus, respectively. They are strong functions of the temperature and the degree of curing and can be expressed in a Prony series as a sum of exponential functions:

(7) $$G\left(\xi \right)=G_{0\;}\left[\;\alpha_{\infty }^{G}+\sum_{i=1}^{n_{G}}\alpha_{i}^{G}\exp\left(-\frac{\xi }{\tau_{i}^{G}}\;\right)\right]$$

(8) $$K\left(\xi \right)=K_{0\;}\left[\;\alpha_{\infty }^{K}+\sum_{i=1}^{n_{K}}\alpha_{i}^{K}\exp\left(-\frac{\xi }{\tau_{i}^{K}}\;\right)\right]$$

G_0_ and K_0_ are the unrelaxed shear and unrelaxed bulk moduli (glassy moduli), while G_∞_ and K_∞_ are the fully relaxed shear and bulk moduli (rubbery moduli). It is assumed that the shear rubbery moduli are independent of the degree of conversion, and their value is near zero [33].

The bulk modulus K is assumed constant in time. In most cases, its relaxation can be discounted [41].

$\alpha_{\infty }^{G}$ is defined as follows:

(9) $$\alpha_{\infty }^{G}=\frac{G_{0}-G_{\infty }}{G_{0}}$$

ξ is the reduced time and can be expressed as:

(10) $$\xi =\int_{0}^{t}\frac{1}{a(T,\alpha )}\;dt^{\prime }\;\;\;\;\;$$

where *a*(*T*,*α*) is the temperature–degree of conversion shift factor.

The effects of curing on viscoelastic properties are considered in the shift factor’s expression, *a*(*T*,*α*). The relaxation time, τ, at a generic temperature and degree of conversion state, in fact, can be expressed as:

(11) $$\tau \left(T,\alpha \right)=\tau_{R}\;/a\left(T,\alpha \right)$$

τ*_R_* is the viscoelastic relaxation time at the reference state assumed to be for each degree of curing at its glass transition temperature, hypothesizing that, independently of the degree of the conversion, the relaxation time does not change at the glass transition temperature.

The last one evolves as a quadratic function of the degree of curing [33].

(12) $$T_{g}\left(\alpha \right)=b_{1}+b_{2}\;\alpha +b_{3}\;\alpha^{2}$$

where b_1_, b_2_ and b_3_ are material constants. Their numerical values are reported in Table 2 At a given degree of conversion, the temperature–degree of conversion shift factor is defined through the following modified TNM [42] equation:

(13) $$a\left(T,\alpha ,T_{f}\right)=exp\left(-\frac{\Delta H\left(\alpha \right)}{R}\;\left(\;\frac{x\left(\alpha \right)}{T}+\frac{1-x\left(\alpha \right)}{T_{f}}-\frac{1}{T_{g}(\alpha )}\;\right)\right)$$

(14) $$\Delta H\left(\alpha \right)=k_{4}+k_{5}\alpha +k_{6}\alpha^{2}$$

(15) $$x\left(\alpha \right)=k_{7}+k_{8}\alpha \;$$

**Figure 1:**
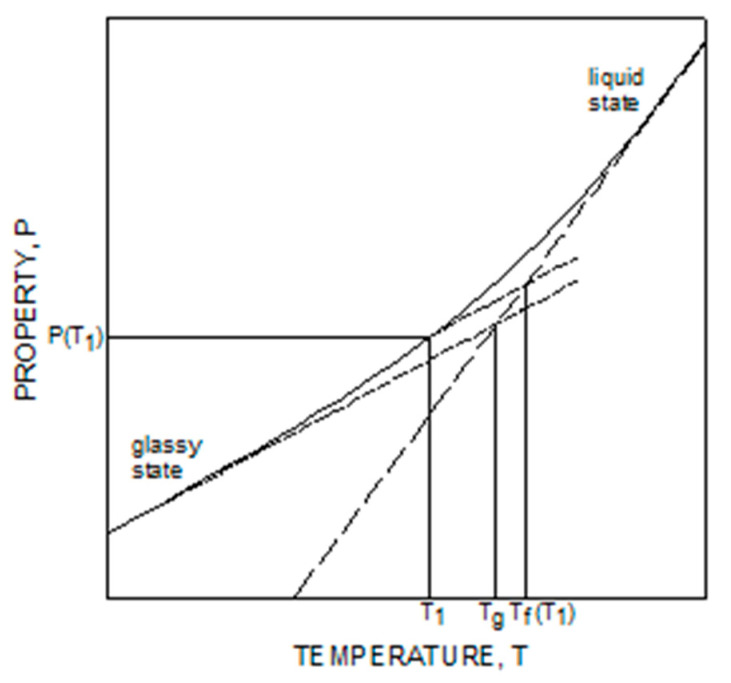
Schematic of a generic property, P, against temperature during the glass transition: Definition of Fictive Temperature.

ΔH is the activation energy, and x is the non-linearity parameter that allows us to distinguish the effect of temperature and the structure on relaxation time. Both parameters are functions of the degree of conversion, k_4_, k_5_, k_6_, k_7_ and k_8_ are material coefficients reported in Table 2. *T_f_* is the fictive temperature, a parameter that univocally identifies the glassy state during the glass transition. The concept of fictive temperature was initially introduced by Tool [43] and can be graphically represented by taking a point in the glassy state and drawing from it a line parallel to the glassy line until it intersects the equilibrium (liquid) line. The abscissa of the intersection is the fictive temperature, as shown in Figure 1, where the evolution of a generic property, P, against the temperature during the glass transition is reported.

Its evolution in time can be evaluated according to the TNM model [42]:

(16) $$T_{f}\left(t\right)=T\left(t\right)-\int_{0}^{t}M(\xi \left(t\right)-\xi \left(t^{\prime }\right))\;\;\frac{dT(t^{\prime })}{dt^{\prime }}dt^{\prime }$$

where ξ(t) is the reduced time. Following Equation (13), it can be expressed as:

(17) $$\xi \left(t\right)=\int_{0}^{t^{\prime }}exp\left[\frac{\Delta H\left(\alpha \right)}{R}\;\left(\;\frac{x\left(\alpha \right)}{T}+\frac{1-x\left(a\right)}{T_{f}}-\frac{1}{T_{g}(\alpha )}\;\right)\right]dt^{\prime }\;$$

M(ξ) is the memory function expressed as:

(18) $$M\left(\xi \right)=\sum_{i=1}^{N_{m}}\alpha_{i}^{M}\;exp(-\frac{\xi }{\tau_{i}^{M}})\;$$

From a physical perspective, it is assumed that the relaxation process can be divided into *N_m_* relaxation processes with different relaxation times and weight factors.

(19) $$\sum_{i=1}^{N_{m}}\alpha_{i}^{M}\;=1\;$$

The implicit and unconditionally stable numerical algorithm proposed by Markovsky and Soules [44] was used for the fictive temperature calculation.

Since the expression proposed for the shift factor is not implemented in Ansys, the shift factor was given through a fictitious Arrhenius equation. The equality between Equation (13) and the Arrhenius expression was imposed:

(20) $$exp\left(\frac{\Delta H\left(\alpha \right)}{R}\left(\frac{x(\alpha )}{T}+\frac{1-x(\alpha )}{T_{f}}-\frac{1}{T_{g}\left(\alpha \right)}\right)\right)=exp\left(\frac{{\Delta H}_{Arrhenius}}{R}\left(\frac{1}{T}-\frac{1}{T_{g}\left(\alpha \right)}\right)\right)$$

The “fictitious activation energy”, Δ*H_Arrhenius_*, that assures the equality of the two expressions can be written as:

(21) $${\Delta H}_{Arrhenius}(\alpha ,T_{f},T)=\frac{\Delta H\left(\alpha \right)\;T\;\left(T_{f}+T_{r}\left(-1+x\right)\right)-T_{g}(\alpha )\;x)}{T_{f}\;(T-T_{g}(a))}$$

The equivalent shift factor can be evaluated at each timestep based on the degree of conversion, the temperature, and the fictive temperature previously calculated.

The free strain *ε_free_* in Equation (6) for the case under study is the sum of the three components: the thermal strain due to temperature variations, the chemical strain due to the crosslinking reaction, and the strain due to the change of glass structure caused by the structural relaxation. The incremental free strain for each timestep can be evaluated as:

(22) $$\Delta \varepsilon_{free}^{k}={{CTE}_{glass}}^{k}\;\left(T^{k}-T^{k-1}\right)+{{CTE}_{liquid}}^{k}\left({T_{f}}^{k}-{T_{f}}^{k-1}\right)-(\alpha^{k}-\alpha^{k-1})\lambda_{max}\;$$

The coefficient of thermal expansion is a linear function of the degree of conversion and it assumes a different value in the glass or liquid state [45]. *λ_max_* is the maximum chemical contraction due to crosslinking network formation.

An equivalent thermal expansion coefficient was given to Ansys to consider the three components of free strain. This equivalent CTE considers both the thermal and chemical strain effects:

(23) $${{CTE}_{eq}}^{k}=\varepsilon_{free}^{k}/(T^{k}-T_{ref})\;$$

where *T_ref_* is the reference temperature for the thermal strain calculation, ε_free_^k^ is the free strain at the timestep *k* and can be evaluated as the sum of free strain increments until time step *k*.

(24) $$\varepsilon_{free}^{k}=\sum_{i=1}^{k}\Delta \varepsilon_{free}^{i}\;$$

### 2.2. FEM Model

**Figure 2:**
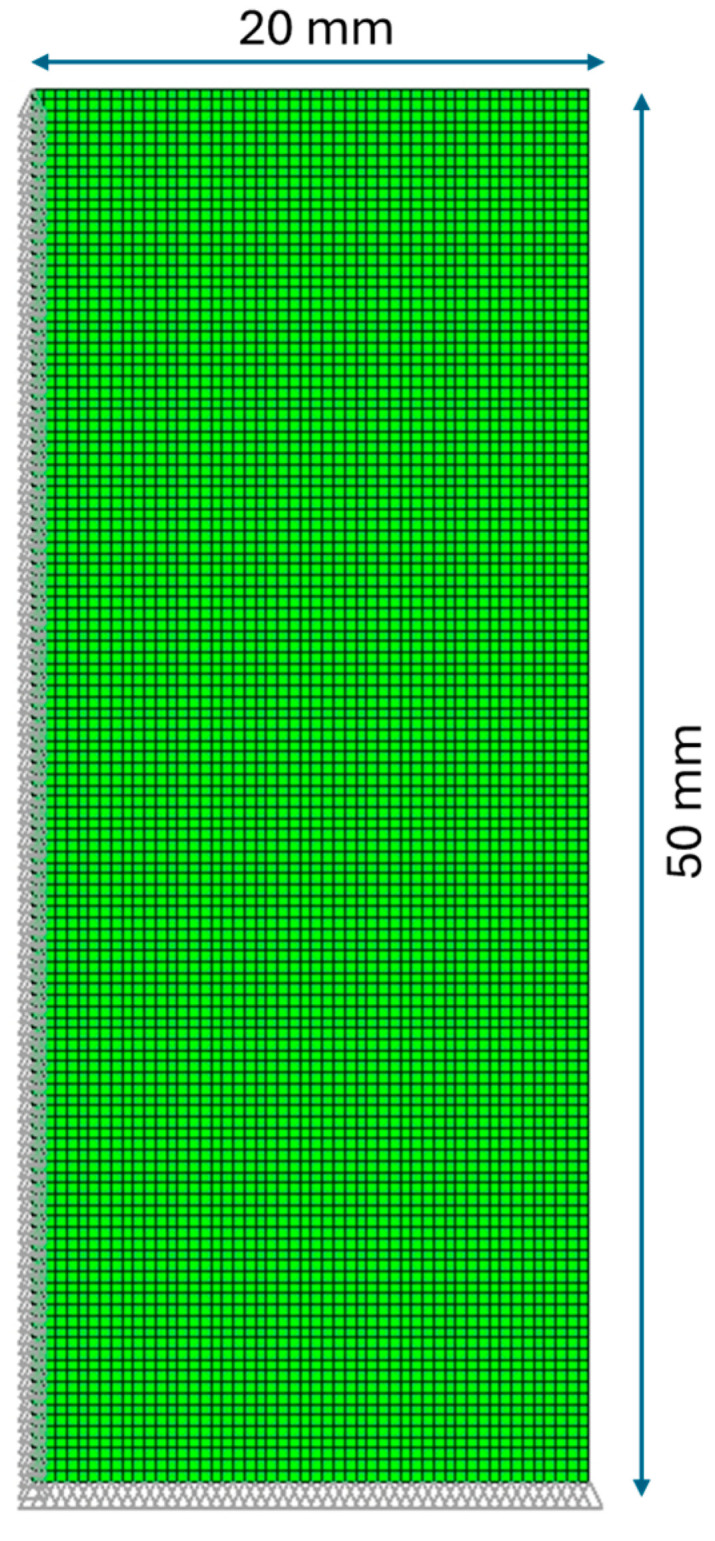
Cylinder’s FEM model.

The case under study is a cylindrical geometry with radius r = 20 mm and height l = 100 mm. The discretized geometry is shown in Figure 2.

**Figure 3:**
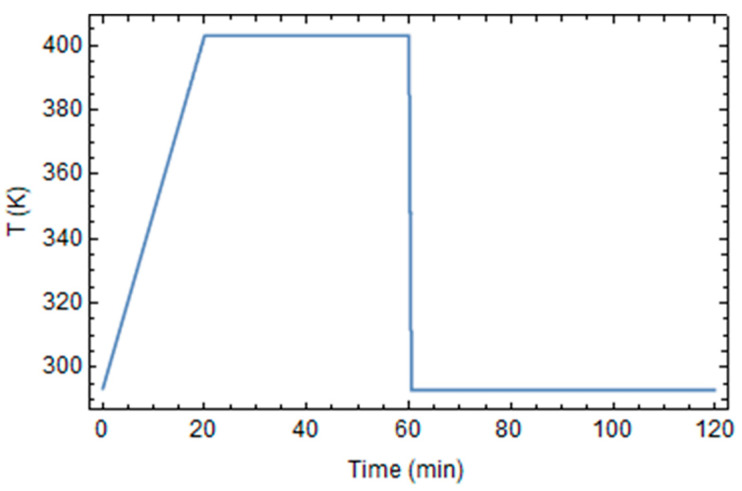
The temperature profile of the cylinder’s external surface.

An arbitral temperature cycle, shown in Figure 3, was applied on the cylinder’s external surface to induce the resin’s crosslinking reaction: the temperature initially varies from the room temperature, T_0_ = 20 °C, to the cure temperature, T*_cure_* = 130 °C, over 20 min; the temperature was kept constant for 40 min and, at the end, the epoxy is rapidly cooled to room temperature in 30 sec. Then, the cylinder was maintained at room temperature for a sufficient time to permit the complete cooling of its inner part.

The convection coefficient h is set equal to 100 W/m^2^K during the entire process. It is a medium value of convention in a fan oven.

Since the problem has axial and transversal symmetry, only a quarter of the geometry is analyzed. Plane elements with an axisymmetric option are used. The element type used for the thermal analysis is PLANE 55, which is a quadrilateral element with four nodes and only a degree of freedom for each node (the temperature), while for the structural analysis, PLANE 182, with four nodes and 2 degrees of freedom for each node (with displacements in the radial and axial directions) is used. The FE model contains 6300 elements, and the element’s characteristic dimension is 0.4 mm. As boundary conditions, on the symmetry lines, the radial displacement of the nodes on the cylinder’s axis and the axial displacement of the nodes on the cylinder’s central transversal line are constrained.

Given its liquid-like consistency, epoxy resin cannot build stress at a degree of conversion lower than its gel point. Thus, calculations start at room temperature *T*_0_ = 293 K and α_0_ = 0.3.

The proposed numerical procedure comprises two moduli that must be solved consecutively: the thermal kinetical module and the structural one. In the first module, the thermal and the kinetics problems are solved in a coupled way at each timestep: at first, the local temperature is evaluated by solving the energy balance in the thermal module of Ansys, then the kinetics equations are solved using a homemade routine written in APDL. In particular, the differential equations of kinetics, shown in Equations (2) and (3), are solved using the explicit Euler method, allowing the calculation of the degree of conversion for each node at each timestep. The internal heat generation due to the exothermic reaction at that timestep is calculated based on the knowledge of the rate of conversion and applied as the thermal nodal load at the next timestep. This procedure is iterated for all successive timesteps.

Then, each node’s temperature and degree of conversion profiles are used as input for the structural module, accounting for the material’s mechanical properties. Since the shift factor is a function of the fictive temperature, at first, the fictive temperature is estimated at each timestep for every element, using the numerical algorithm proposed by Soules [44] and explained in the previous section; then, from the information of the temperature and degree of conversion profiles, it is possible to evaluate the resin’s viscoelastic properties allowing for the calculation of residual stresses during the process.

Since the mechanical properties are functions of the degree of curing that has a nonuniform geometrical distribution during the process, a material for each element must be defined, and its material properties must be updated at each timestep as a function of the degree of conversion.

### 2.3. Model Parameters

All model parameters are reported in detail in part I [34] of this work, where the sources and the relative hypothesis are precisely described. In this paragraph, the model input data are briefly summarized.

The thermal and kinetic parameters are reported in Table 1.

To characterize the epoxy’s viscoelastic behavior, we assigned the instantaneous values of two viscoelastic functions and Prony’s coefficients for the shear relaxation referred to the reference state (that is assumed to be at the glass transition temperature for each degree of curing). For the instantaneous value, we assume that the elastic module is 3.2 GPa and the Poisson ratio is 0.35 [33].

The material parameters defining the glass transition temperature (Tg), the activation energy (ΔH) and the non-linearity parameters (x) for evaluating the shift factor are presented in Table 2.

The Prony coefficients for the shear relaxation modulus are reported in Table 3, whereas those of the memory function are reported in Table 4.

## 3. Results and Discussion

**Figure 4:**
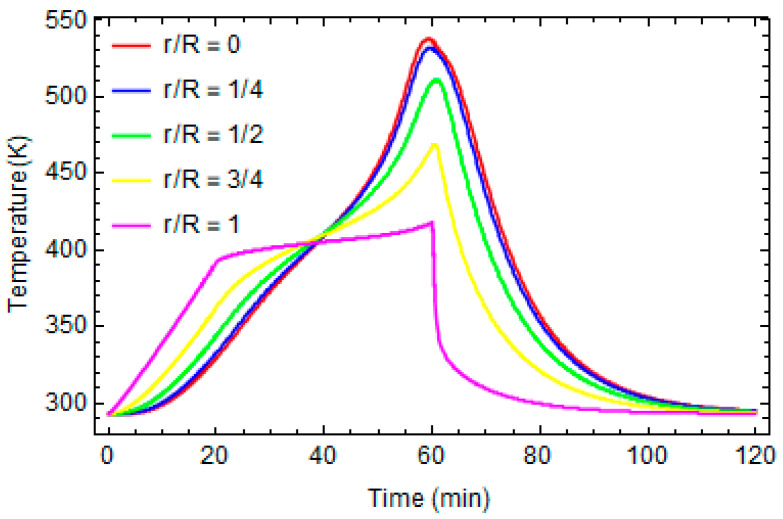
Temperature profiles at different distances from the center of cylinder.

**Figure 5:**
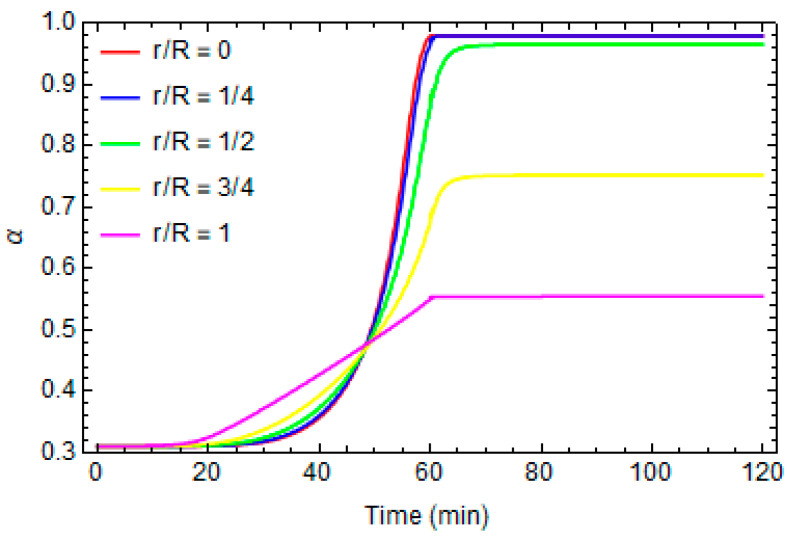
Degree of conversion profiles at different distances from the center of cylinder.

Figure 4 and Figure 5 report the evolution of the local temperature and the degree of conversion at different distances from the center of the cylinder, respectively. In the inner part, the temperature is higher than in the proximity of the external surface because the system is more thermally isolated, and consequently, the effects of the exothermic reaction are more significant. The maximum temperature during the process is about 530 K.

The crosslinking reaction is faster when the local temperature is higher and the inner parts are fully cured at the end of the process (α = 0.98), while the areas near the external surface do not reach the crosslinking reaction’s end.

**Figure 6:**
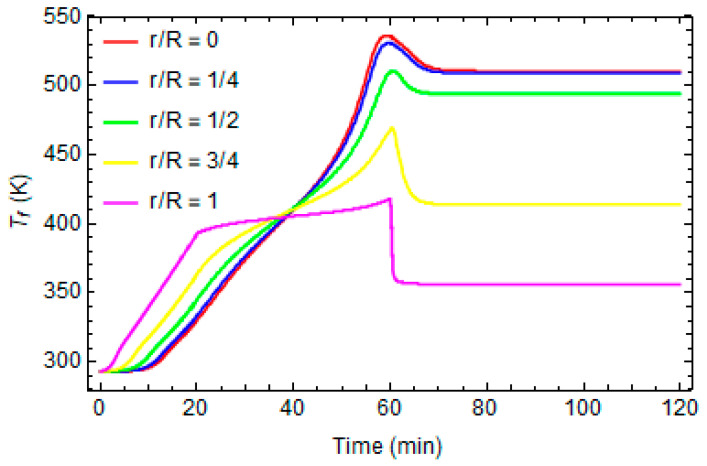
Fictive temperature profiles at different distances from the center of the cylinder.

Consequently, at the end of the curing, the glass transition temperature is not uniform, as shown in Figure 6, where the fictive temperature profiles at different distances from the center of cylinder are reported. In the first phases of the curing, the fictive temperature overlaps the local temperature until cooling to room temperature, when the material passes from liquid to glassy state and the fictive temperature equals the local glass transition temperature. The last one assumes different values at different distances from the cylinder’s center because the degree of conversion is not uniform and because different points suffer different cooling rates.

The origin of residual stresses is caused by the non-uniform temperature distribution during the curing, which produces different degrees of conversion on the geometry. Since the mechanical properties are a function of the temperature and the degree of curing, a mismatch of mechanical properties in the component causes stress build-up.

**Figure 7:**
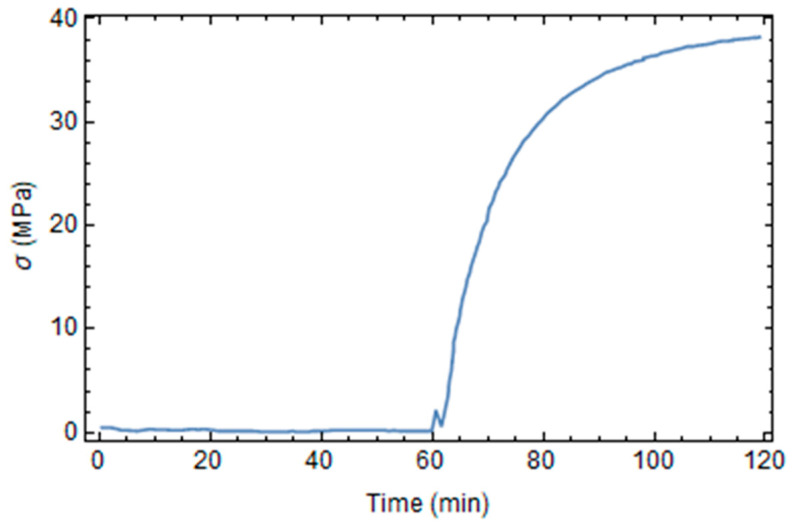
Evolution of von Mises maximum stress during the process.

Residual stresses arise, especially during cooling at room temperature, as shown in Figure 7. In fact, during the first phases of the process, the material is at a high temperature and a low degree of conversion, so the relaxation of stress is almost instant, as explained in the previous section. Residual stresses are a consistent portion of resin’s nominal strength.

**Figure 8:**
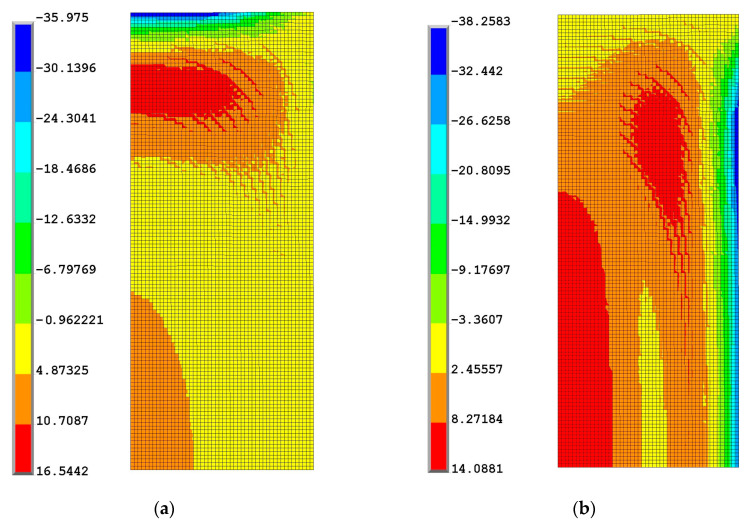
Contour plot of the radial (**a**) and axial (**b**) components of stress at the end of the process.

**Figure 9:**
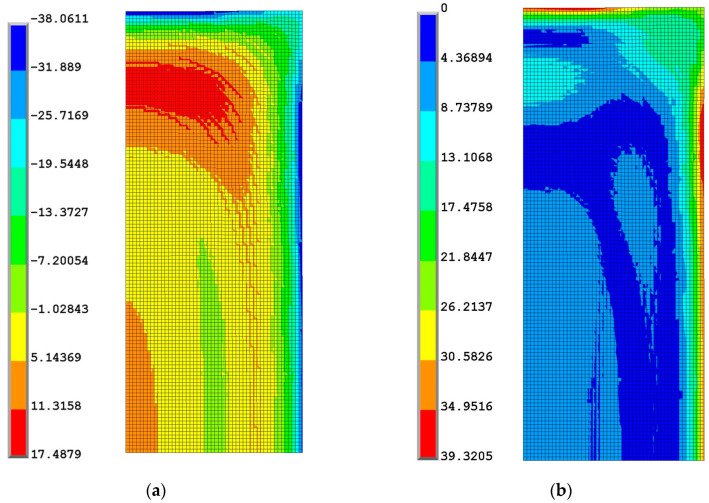
Contour plot of the hoop component of stress (**a**) and of the von Mises stress (**b**) at the end of process.

At the end of the high-temperature phase, the external surface reaches a lower degree of conversion than the core, so the coefficient of thermal expansion is higher, and the glass transition temperature is lower than in the inner part. During the cooling to room temperature, the external surface’s temperature decreases more rapidly than the inner part; the contraction of the areas near the external surface causes a stretching of the core that undergoes a tensile state, as shown in Figure 8 and Figure 9, where the stress component contour plots at the end of curing are reported.

A parametric analysis is performed to understand the effect of the heat transfer coefficient h and the cylinder’s radius r on the residual stresses.

It is noted that the amount of stress is higher when the Biot number increases, and the relationship between the maximum stress, σ, and the logarithmic of the Biot number, Bi, can be expressed as a stretched exponential:

(25) $$\sigma =k\;exp(-{\left(\frac{log(Bi)}{\delta }\right)}^{ϒ})$$

(26) $$Bi=\frac{hr}{k}\;\;\;$$

where k, ϒ, and δ are fitting parameters obtained, minimizing the interpolation error.

**Figure 10:**
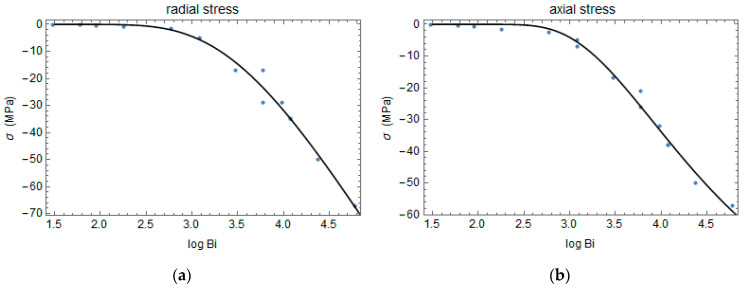
Radial (**a**) and axial (**b**) stress versus the logarithmic of the Biot number.

**Figure 11:**
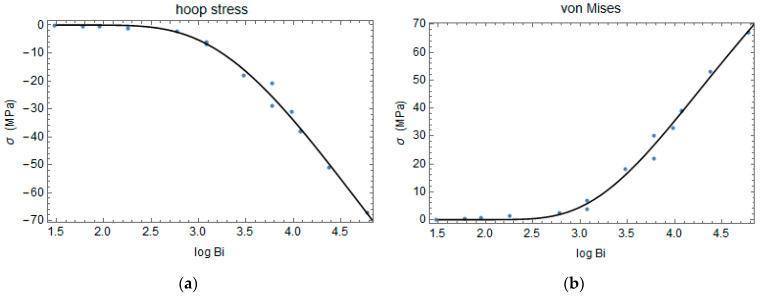
Hoop (**a**) and von Mises (**b**) stress versus the logarithmic of the Biot number.

The relationships between residual stress components and the Biot number are plotted in Figure 10 and Figure 11.

## 4. Conclusions

This work investigated the effect of the curing process on the residual stresses developing in a polymeric part. In particular, a numerical procedure was proposed and implemented in the Ansys environment to predict the residual stresses in an epoxy cylinder, considering the coupling between the curing, the viscoelasticity, and the structural relaxation for the first time.

Results show that residual stresses arise, especially during cooling at room temperature, and are a consistent portion of the resin’s nominal strength. Before the cooling, the stresses are negligible because relaxation occurs almost instantly. In addition, a parametric analysis highlights that the amount of stress scales as a stretched exponential logarithmic of the Biot number.

## Figures and Tables

**Table 1:** Thermal and kinetic parameters for epoxy 3501 [40].

**A_1_**	2.102 × 10^9^	min^−1^
**A_2_**	−2.014 × 10^9^	min^−1^
**A_3_**	1.960 × 10^5^	min^−1^
**ΔE_1_**	8.07 × 10^4^	J/mol
**ΔE_2_**	7.78 × 10^4^	J/mol
**ΔE_3_**	5.66 × 10^4^	J/mol
**H_r_**	473.16	kJ/ Kg
**R**	8.314	J/Kg mol
**ρ**	1200	Kg/m^3^
**c_p_**	1260	J/Kg K
**k**	0.167	W/m K

**Table 2:** Material parameters defining the glass transition temperature (Tg), the activation energy (ΔH) and the non-linearity parameter (x).

**b1**	284.46
**b2**	−47.33
**b3**	239.4
**k4**	8272
**k5**	−18,541
**k6**	2780
**k7**	0.833
**k8**	0.0374

**Table 3:** Prony’s coefficients for shear relaxation modulus at the reference state [33].

**N**	**τ_G_ (s)**	**α_i_^G^**
**1**	1.75 × 10^−9^	0.059
**2**	1.75 × 10^−7^	0.066
**3**	1.09 × 10^−5^	0.083
**4**	6.60 × 10^−4^	0.112
**5**	1.70 × 10^−2^	0.154
**6**	4.76 × 10^−1^	0.262
**7**	1.17 × 10^1^	0.184
**8**	2.00 × 10^2^	0.049
**9**	2.95 × 10^4^	0.025

**Table 4:** Prony’s coefficients of memory function are at the reference state.

**N**	**τ_M_ (s)**	**α_i_^M^**
**1**	1.21 × 10^−6^	0.0062
**2**	2.60 × 10^−5^	0.0072
**3**	5.60 × 10^−4^	0.0175
**4**	1.21 × 10^−2^	0.0390
**5**	2.60 × 10^−1^	0.0856
**6**	5.60	0.1730
**7**	1.21 × 10^2^	0.2950
**8**	2.60 × 10^3^	0.298
**9**	5.60 × 10^4^	0.0785

## Data Availability

The raw data supporting the conclusions of this article will be made available by the authors on request.
